# Status of age-friendly city indicators in Iran cities: a systematic review

**DOI:** 10.1186/s12877-024-05021-1

**Published:** 2024-05-09

**Authors:** Fatemeh Fallahi, Mohsen Adib-hajbaghery, Azade Safa

**Affiliations:** Trauma Nursing Research Center, Kashan University of Medical Sciences, Kashan, Iran

**Keywords:** Age-friendly city, Iran, Systematic review

## Abstract

**Background:**

The global phenomena of an increasing older population within the total population and the rise in urban older residents have prompted numerous studies on the indicators of an age-friendly city in various Iranian cities. The insights obtained from these studies can aid policymakers in promoting social justice for older adults. Thus, this study aimed to investigate the status of age-friendly city indicators across different cities in Iran.

**Method/design:**

A systematic review was conducted by searching for studies in Persian and English databases until March 2024, including Pubmed, Web of Science, Scopus, Cochrane, Google Scholar, and ScienceDirect, using keywords such as “age-friendly”, “elderly-friendly”, “cities”, “older adults”, “aging”, “elderly”, “indicators”, “components”, “criteria”, “features”, “characteristics”, “indexes”, “Iran”, and “urban space”, along with their MeSH equivalents, employing “AND” and “OR” operators. Additionally, Persian databases such as Magiran and SID were extensively searched using keywords like “elderly-friendly”, “city”, “urban spaces”, “Iran”, “indicators”, “components”, “features”, and “criteria”. The references of the final articles were also examined to ensure search accuracy. The results from the studies on the indicators of an age-friendly city were reviewed, summarized, and ultimately reported.

**Results:**

The initial search yielded 2857 articles, of which 34 were included in the systematic review. Only two studies addressed the indicators based on the needs of the older adults, with the majority reporting unfavorable urban conditions for the older adults. Despite these unfavorable conditions, the index of open spaces and buildings received the highest rank among the examined indicators. However, two indicators—respect for older adults, social acceptance, civic participation, and employment— did not perform well in any study.

**Conclusion:**

The findings indicated that Iran failed to meet the required standards for age-friendly cities. Therefore, it is recommended that policymakers in the field of geriatric health take measures to identify and mitigate environmental risk factors for older adults.

**Trial Registration:**

This systematic review was registered on the Prospero database with the number CRD42023475657 on date 8 November 2023.

## Background

**Fig. 1:**
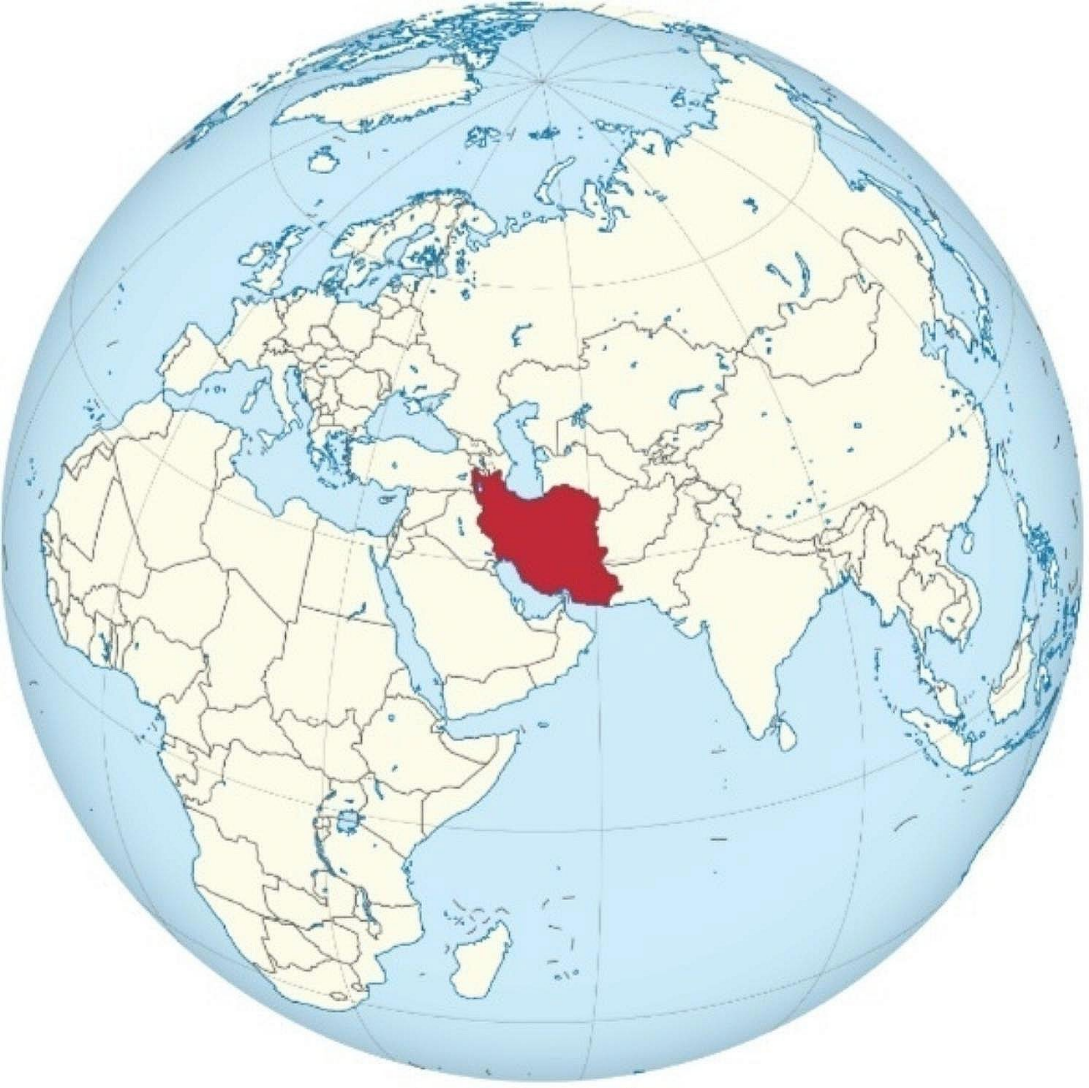
Iran’s location on the globe. (From: https://en.wikipedia.org/wiki/File:Iran_on_the_globe_%28Iran_centered%29.svg)

The global community is currently grappling with two significant phenomena: the increasing proportion of older adults within the overall population and the rise in the urban older population. According to the World Health Organization (WHO), the rate of population aging is accelerating at an unprecedented pace, with projections indicating that between 2015 and 2050, the global population aged 60 and above will surge from 12 to 22% [1]. In Iran, the 2016 census revealed that the older population exceeds 7 million people (about 9%), and it is estimated that this percentage will reach more than 10% in 2050 [2, 3]. Decrease in fertility rate, increase in life expectancy, technological advances and economic and social development of the society in recent years have caused a change in the demographic structure of Iran’s population and an increase in the number of older people in this country [4]. With a land area of 1,648,195 square kilometers, Iran is the second-largest country in the Middle East (Fig. 1). It is situated at the crossroads of Asia, Europe, and Africa, home to over 89 million people [5, 6]. Due to its rich and ancient culture, Iran annually attracts a substantial number of foreign tourists, with a significant portion of them being older adults [7]. Concerns arise due to changes in the population’s structure that affect this age group.

In the past two decades, the simultaneous phenomena of population aging and urbanization have brought attention to the concept of an “age-friendly city.” An age-friendly city is a city where older people have equal rights as citizens of other age groups. In such a city, urban standards and designs are respected in a way that creates suitable opportunities for the participation and security of older adults. These cities are designed for older adults with various capacities [8]. This concept emphasizes active aging to enhance health and security among older adults, ultimately improving their quality of life. It is crucial to identify the capabilities and needs of older adults, providing them with the opportunity for effective participation and increased social presence [9]. An age-friendly city characterized by urban facilities and infrastructures that enable active aging, allowing older adults, regardless of their abilities, to actively participate in society. In contrast, inadequate urban spaces and a lack of support for active aging can compromise the health of the entire society, leading to feelings of fear, social isolation, depression, and increased disability among older adults [10].

In 2007, the WHO introduced eight domains for an age-friendly city, including open spaces and public buildings, transportation, housing, social participation, social respect and acceptance, civic participation and employment, communication and information, and social support and health services. Assessing a city’s status based on these indicators provides insight into how age-friendly it is [11]. Several studies have been conducted on age-friendly cities in various Iranian cities, examining different indicators. Some studies have assessed all indicators [12], while others have focused on specific indicators in a city [13] or a particular urban district [14]. The results of these studies have varied across different regions. For example, a study in Mashhad reported relatively favorable conditions for older adults in that city [15], whereas another study found that the criteria for an age-friendly city in Karaj did not meet the standard [16]. Analyzing these studies allows for a partial understanding of Iran’s present status regarding the indicators of an age-friendly city.

Given the increasing urbanization and the growing influence of surroundings on older adults, ensuring the safety and comfort of older adults has become more important than ever. Therefore, it is necessary to assess the conditions of society and cities and adapt urban areas to meet the needs of older adults, facilitating their presence and activity in the country.

### Objectives

Researchers have undertaken a systematic review to address the question: What is the status of Iran in terms of the indicators of an age-friendly city?

### Method/design

This systematic review study examined the status of the indicators of an age-friendly city in Iranian cities. This systematic review has adhered to the Preferred Reporting Item for Systematic Review and Meta-Analyses (PRISMA 2020) guideline and checklist [17]. It was registered on the Prospero database with the number CRD42023475657 on November 8, 2023. Articles related to the indicators of an age-friendly city in Iran collected up to March 1, 2024.

### Inclusion and exclusion criteria

**Fig. 2:**
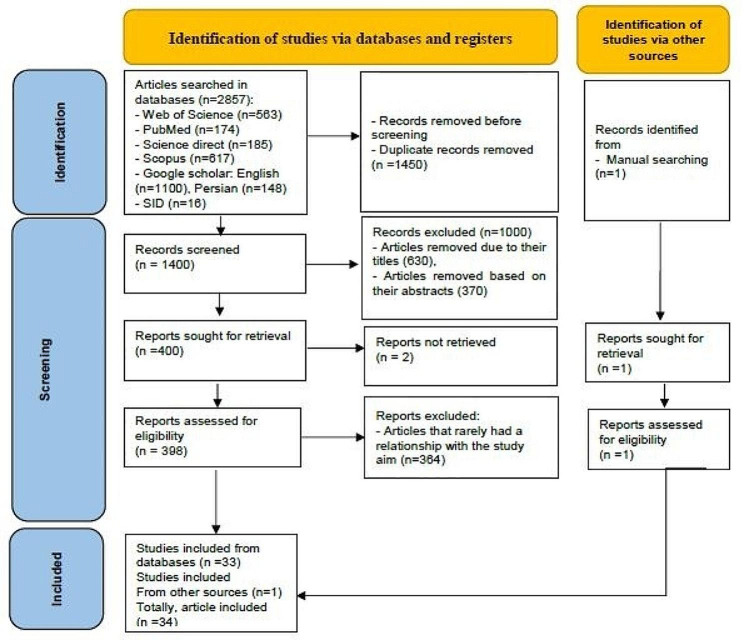
The process of selecting articles and reviewing literature

Inclusion criteria comprised articles in Persian or English, access to the full text, publication in scientific journals, and descriptive or qualitative studies with keywords or their equivalents present in the title or abstract. The exclusion criterion involved articles that did not provide data on any of the indicators of an age-friendly city in Iran. Articles that were unavailable in full-text were excluded. A total of 34 studies were examined (Fig. 2).

### Search strategy

In the search strategy, various English databases and search engines such as Pubmed, Web of Science, Scopus, Cochrane, Google Scholar, and ScienceDirect were utilized. The search included keywords such as “Age-friendly”, “elderly-friendly”, “cities”, “older adults”, “Aging”, “elderly”, “indicators”, “components”, “criteria”, “features”, “characteristics”, “indexes”, “Iran” and “urban space” along with their MeSH equivalents, using “AND” and “OR” operators. Additionally, Persian databases, including Magiran and SID were extensively searched using keywords such as “age-friendly”, “city”, “urban spaces”, “Iran”, “indicators”, “components”, “characteristics” and “criteria”. The references of the final articles were also checked to ensure the search accuracy. Moreover, to find any additional related works that were missed in the search process, a manual search was also done in the databases.

**Fig. 3:**
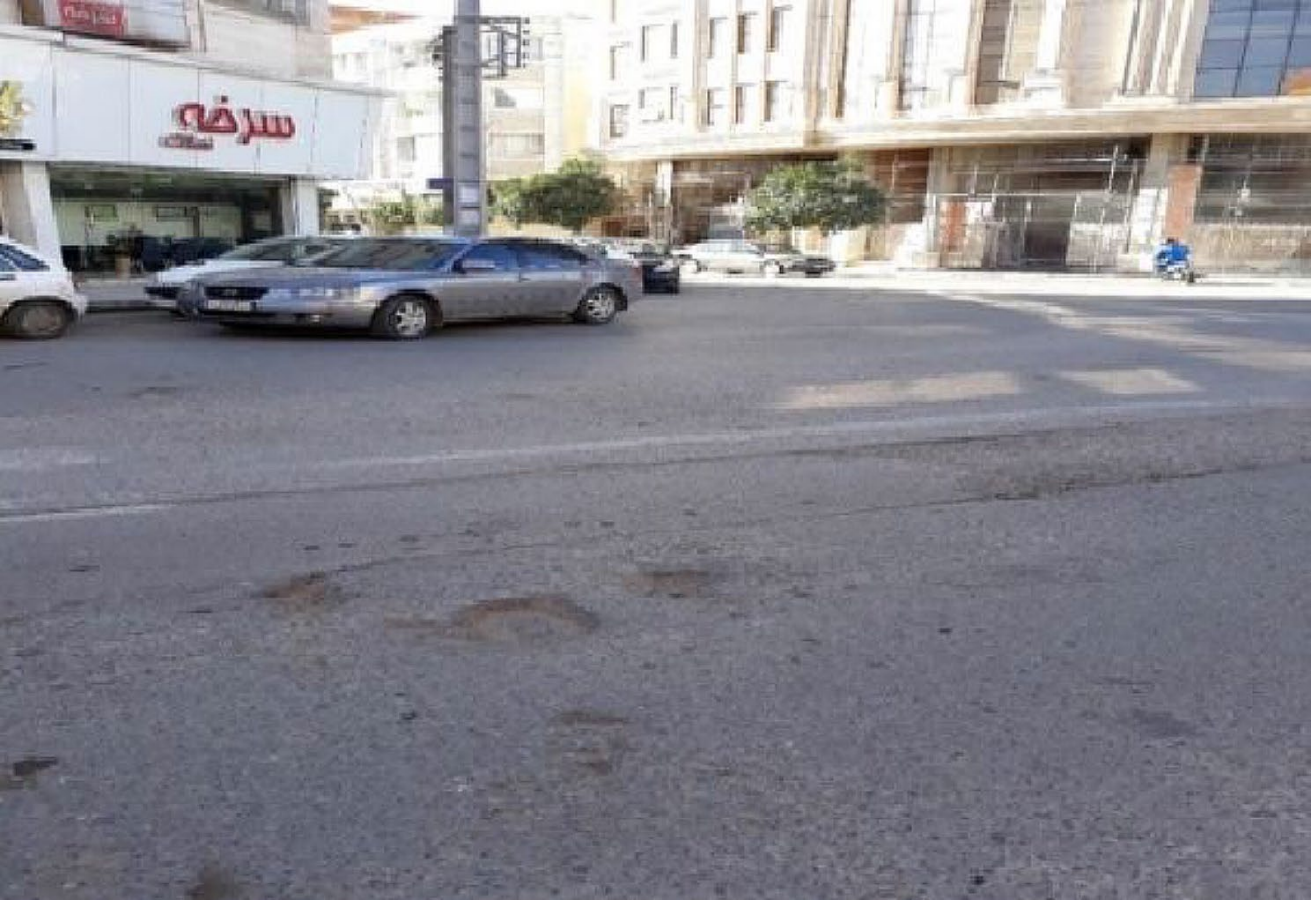
A sample of inappropriate traffic design. (From: Ghaffari Gilandeh A, Mohammadi C, Davari E. Evaluating elderly-friendly city indicators (A case study of Sari). Environ Based Territorial Planning. 2022;15(56):1–25.)

**Fig. 4:**
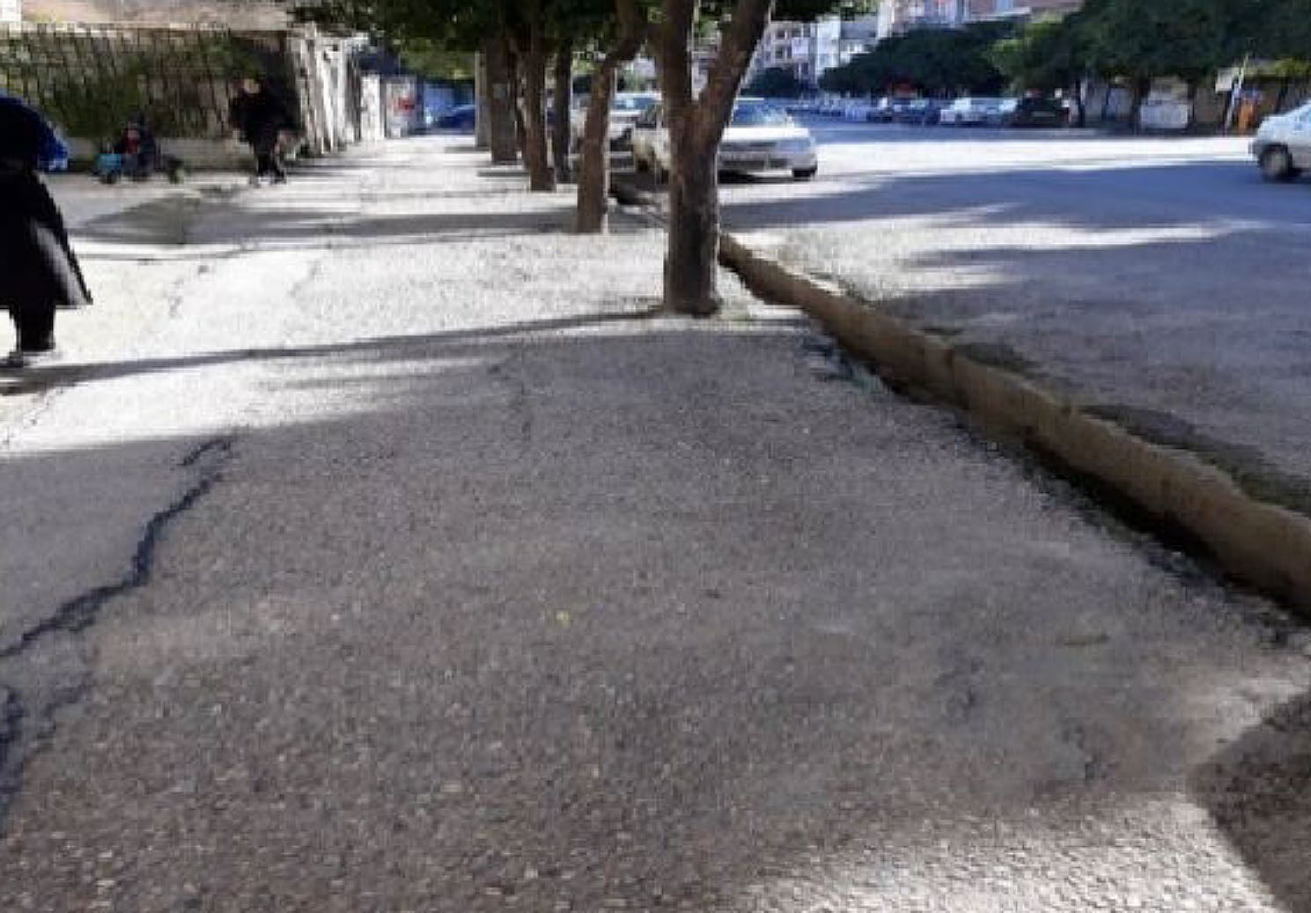
A sample of sidewalks with unsafe flooring. (From: Ghaffari Gilandeh A, Mohammadi C, Davari E. Evaluating elderly-friendly city indicators (A case study of Sari). Environ Based Territorial Planning. 2022;15(56):1–25.)

**Fig. 5:**
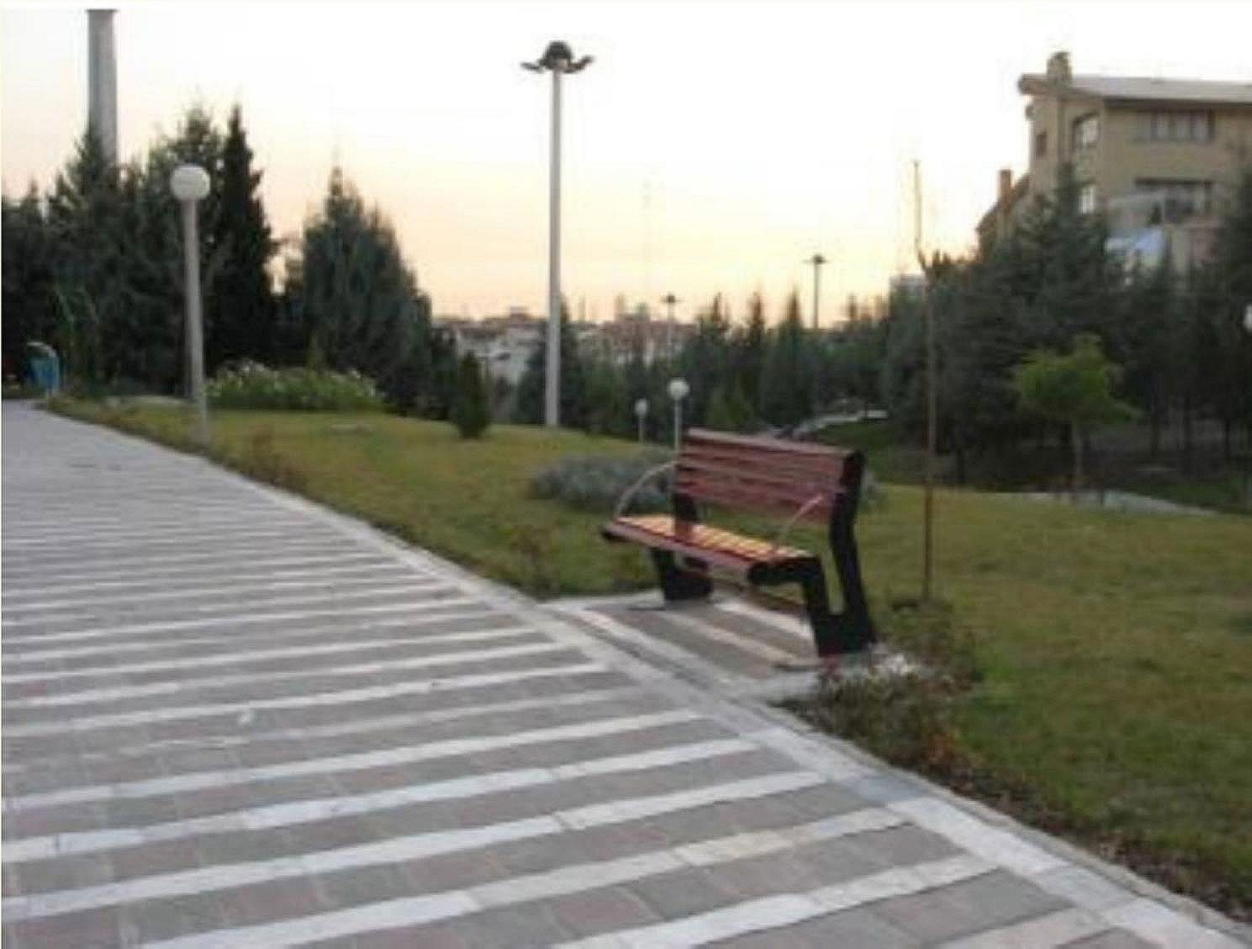
A sample of lack of non-standard furniture and equipment for older adults. (From: Sharqi A, Zarghami E, Olfat M, Salehi Kousalari F. Evaluating status of global indices of age-friendly city in Tehran metropolis (AFC). J Urban - Regional Studies and Research. 2016;8(28):1–22.)

The search strategy in different databases could not be implemented similarly due to limitations in some Persian databases, which are not sensitive to Boolean operators (NOT, AND, OR). For instance, in certain Persian databases, like SID and Magiran advanced search features are limited, and the sensitivity to Boolean operators is limited. Consequently, the search was restricted to keywords that produced the most favorable outcomes. The ScienceDirect database does not offer advanced search capabilities like other databases. Search boxes have character limitations, accepting only up to eight characters for searching. As a result, search results were either very limited or excessively broad. Therefore, the search mode that yielded the highest number of relevant articles was selected.

However, for other English databases, keywords were standardized using MeSH (Medical Subject Headings) terms, and they were utilized for the search (Table 1).

**Table 1:** Search strategy in databases

Database	Strategy	Number of articles
Web of Science	“age-friendly” OR “elderly-friendly” OR “age-friendliness” OR “age-inclusive” OR “age-appropriate” OR “senior-friendly” OR “geriatric-friendly” OR “elder-friendly” (Topic) AND cities OR “urban space” OR city OR town OR community OR communities OR environment OR neighborhood OR neighbourhood (Topic) AND standards OR indicators OR components OR criteria OR features OR characteristics OR indexes (Topic) AND aging OR elderly OR “older adults” (Topic) and English (Languages) and Early Access or Proceeding Paper or Meeting Abstract or Book Review or Retracted Publication or Book Chapters or Editorial Material (Exclude – Document Types)	563
Pubmed	(((“age-friendly“[Title/Abstract] OR “elderly-friendly“[Title/Abstract] OR “age-friendliness“[Title/Abstract] OR “age-inclusive“[Title/Abstract] OR “age-appropriate“[Title/Abstract] OR “senior-friendly“[Title/Abstract] OR “geriatric-friendly“[Title/Abstract] OR “elder-friendly“[Title/Abstract]) AND (cities[Title/Abstract] OR cities[Mesh] OR"urban space“[Title/Abstract] OR city[Title/Abstract] OR town[Title/Abstract] OR community[Title/Abstract] OR communities[Title/Abstract] OR environment[Title/Abstract] OR neighborhood[Title/Abstract] OR neighbourhood[Title/Abstract])) AND (standards[Title/Abstract] OR standards[Mesh] OR indicators[Title/Abstract] OR components[Title/Abstract] OR criteria[Title/Abstract] OR features[Title/Abstract] OR characteristics[Title/Abstract] OR indexes[Title/Abstract])) AND (aging[Title/Abstract] OR aging[Mesh] OR elderly[Title/Abstract] OR “older adults“[Title/Abstract])	174
Cochrane	(“age-friendly” OR “elderly-friendly” OR “age-friendliness” OR “age-inclusive” OR “age-appropriate” OR “senior-friendly” OR “geriatric-friendly” OR “elder-friendly”):ti, ab, kw AND (cities OR “urban space” OR city OR town OR community OR communities OR environment OR neighborhood OR neighbourhood): ti, ab, kw AND (standards OR indicators OR components OR criteria OR features OR characteristics OR indexes): ti, ab, kw AND (aging OR elderly OR “older adults”):ti, ab, kw	0
ScienceDirect	(“age-friendly” OR “elderly-friendly” OR “age-friendliness” OR “age-inclusive” OR “age-appropriate” OR “senior-friendly” OR “geriatric-friendly” OR “elder-friendly”) AND (aging) Title, abstract, keywords: AND (cities) AND (standards OR indicators OR components OR criteria OR features OR characteristics OR indexes)	185
Scopus	( TITLE-ABS-KEY ( “age-friendly” OR “elderly-friendly” OR “age-friendliness” OR “age-inclusive” OR “age-appropriate” OR “senior-friendly” OR “geriatric-friendly” OR “elder-friendly” ) AND TITLE-ABS-KEY ( cities OR “urban space” OR city OR town OR community OR communities OR environment OR neighborhood OR neighbourhood ) AND TITLE-ABS-KEY ( standards OR indicators OR components OR criteria OR features OR characteristics OR indexes ) AND TITLE-ABS-KEY ( aging OR elderly OR “older adults” ) ) AND ( LIMIT-TO ( LANGUAGE, “English” ) OR LIMIT-TO ( LANGUAGE, “Persian” ) )	617
Google scholar	“Age-friendly” OR “elderly-friendly” AND cities OR “urban space” AND indicators OR components OR criteria OR features OR characteristics OR indexes AND aging OR elderly OR “older adults” AND Iran	1100
Google scholar with Persian words	“ Age-friendly " AND Component OR Feature OR Indicator OR Criterion AND City AND older adults	148
SID *	“Age-friendly city index”	16
Magiran *	“ Age-friendly " AND Component OR Feature OR Indicator OR Criterion AND City	54

* In Persian databases, the words were searched in Persian

### Study screening

In the first step, the search results were entered into Endnote X9. Then duplicate articles are removed. The articles were initially reviewed based on their titles and then their abstracts. Then the full text of 400 articles was retrieved. Of these, 5 articles were retrieved by sending an email to the author. However, the full text of two articles could not be retrieved due to the author’s lack of response, and it was removed from the article screening process. Subsequently, the full texts of the remaining articles were examined after removing the irrelevant ones. Of the 398 articles, finally, 34 studies entered into the final review.

The results section of these selected articles (34 articles) was examined. No automation tools were utilized in the article screening process; all screening steps, as well as data analysis, were conducted manually. Two researchers carried out all these steps. Any conflicts among the reviewers were resolved through discussion and consulting a senior team member.

### Data extraction and synthesis

Data was extracted to find an overview of the studies’ contents, i.e., study design, settings and study results. A checklist containing authors’ specifications, publication year and type of research, study objective, sample size, data collection method, and main results was utilized to extract data. The data were gathered and synthesized narratively from the data presented in the results and discussion of the articles reviewed.

### Quality assessment

The quality of observational articles was evaluated using the STROBE checklist [18], which includes 22 items across six general sections: title and abstract, introduction, methods, results, discussion, and other information. The maximum score on this checklist is 22 and the minimum is 0. Articles that scored 0–7, 8–14, and 15–22 are considered as being of low, moderate, and high quality, respectively. Those of low quality were excluded from the study.

For qualitative articles, the COREQ checklist [19] was employed, consisting of a 32-item checklist and three main domains: research team and reflexivity, study design and data analysis and reporting. The range of scores for this checklist is 0 to 32. Articles that scored 0–10, 11–21, and 22–32 were considered as being of low, moderate, and high quality, respectively. Articles deemed low quality were excluded from the study.

All steps of searching, quality assessment of articles, and data extraction were performed by two researchers (A.S. and M.A.H) to mitigate bias, and in cases of disagreement, a third researcher (F.F) was consulted.

### Data analysis

The results obtained from the review of full-text articles were analyzed and subsequently reported. A deductive, directed qualitative content analysis approach was used using the method suggested by Hsieh and Shannon (2005) [20]. Therefore, according to the purpose of the research and the criteria of an age-friendly city [11], a structured analysis matrix, including the characteristics and concepts of an age-friendly city, was compiled. Then, the findings from the results and discussion of the articles were extracted and placed in their proper positions in the analysis matrix.

## Results

From all the obtained articles, the full texts of 34 articles related to the study’s aim were examined, and relevant data was extracted. Researchers in the fields of geography, urban planning, and architecture conducted most of these studies, while other studies were carried out by researchers in the fields of health and management, geriatric health, social welfare, and nursing (Table 2).

**Table 2:** Studies examining the status of age-friendly city indicators in Iranian cities

Author’s (year)	City	design	objectives	Sampling/ instrument	Findings
Ghaffari Gilandeh 2022 [30]	Sari	Descriptive	Examining Urban Spaces for Older People Living: Emphasizing the Eight Standard Indicators of an Age-Friendly City	A total of 383 older adults were selected using the convenience sampling method and surveyed using a researcher-made questionnaire.	The mean scores of all indicators were below average. Among these indicators, the social support and health service index had the lowest score.
Estebsari et al. 2021 [13]	Tehran	Descriptive	Evaluation of Transportation Infrastructure and Urban Space of Tehran based on The Indicators of Age Friendly.	A total of 418 older adults were randomly selected and surveyed using the WHO questionnaire.	Among the four investigated indicators, the highest and lowest mean scores were related to the housing index and the health services index, respectively.
Barzanjeh Atri 2022 [24]	Tabriz	Descriptive	Elderly’s Viewpoints on Components of Age-Friendly Cities	A total of 351 older adults were randomly selected and surveyed using the WHO questionnaire.	None of the four health-related components were within the standard range. The highest mean score was assigned to the health and treatment component.
Karami et al. 2022 [25]	Kermanshah	Descriptive	An Age-Friendly City Indicators of Kermanshah Based on WHO Model	A total of 384 older adults were selected using the census method and 36 urban managers were randomly selected and surveyed using the WHO questionnaire.	The overall score of the city was 2.33 from the point of view of the older adults and 2.55 from the point of view of the managers (from 5). There was a significant difference in most indicators between the views of the older adults and managers.
Morowatisharifabad et al. 2022* [12]	Kashan	Descriptive	Age-Friendly City Indicators from the Viewpoint of Older Adults in Kashan City, Iran	A total of 379 older adults were randomly selected and surveyed using a standard questionnaire.	The health and treatment index obtained the highest mean score, while the communication index obtained the lowest mean score.
AhmadiTeymourlouy et al. 2019* [40]	Tehran	Descriptive	Assessing the suitability of the design, safety and physical environment of hospitals for elderly: a case study in Iran	Six hospitals in Tehran were observed using the Age-Friendly Health Care Centers Toolkit of WHO and a standard checklist.	The majority of the hospitals exhibited relatively good safety in the physical environment, but they showed poor resource management and provision of special programs and healthcare systems for older adults.
Nazm Far et al. 2023 [31]	Babool	Descriptive	Assessing and Evaluating the Effects of Urban livability on the Realization of an Elderly-Friendly City	A total of 384 older adults were randomly selected and surveyed using a researcher-made questionnaire.	Out of 12 districts of Babol, only district 4 was elderly-friendly. Among the indicators of the age-friendly city, the index of public spaces and buildings with a weight of 0.2160 won the first place.
Gholami et al. 2022 [34]	Borazjan	Descriptive	Measuring and Evaluating the Indicators of an Elderly-Friendly City	A total of 368 older adults were chosen through convenience sampling and surveyed through a researcher-developed questionnaire to assess seven indicators of an age-friendly city.	The mean scores of all indicators were below average. The lowest was linked to the index of respect for older adults.
Nazmfar et al. 2018 [26]	Bokan	Descriptive	The Feasibility Study of Age-Friendly City in Iranian Cities	A sample of 100 citizens was selected using the convenience sampling method and surveyed using a standard questionnaire to assess the indicators of an age-friendly city.	The mean scores of all indicators fell below the standard. The highest score was associated with the social index, while the lowest score was linked to the communication index.
Ahmadi Teymourlouy et al. 2015* [39]	Tehran	Descriptive	Towards age-Friendly Hospitals in Developing Countries: A Case Study in Iran.	A total of 26 hospitals in Tehran were selected using the convenience sampling method and assessed using a checklist across three dimensions: information and education, management systems, and physical environment and access.	The majority of the hospitals exhibited favorable physical environments and access to public transportation. However, the healthcare-educational programs for older adults, the interaction of the medical staff with old patients, and their prioritization systems were not satisfactory.
Heidari et al. 2021 [48]	Zanjan	Descriptive	Monitoring the Theory of the Right to the City in Reproducing Age–Friendly Space of the City	A total of 170 older adults were selected using a cluster sampling method and assessed using a questionnaire to examine the components and dimensions of the Right to the City theory.	The area under study had a lower-than-standard status based on the theory (2.94). The mean score for the right to use urban space was 3.01, indicating an unfavorable condition.
Samei et al. 2022 [27]	Tabriz	Descriptive	Viewpoints of Older People toward the Features of Age-Friendly Communities: Map for Charting Progress in Tabriz, Iran	A random sample of 384 older adults was assessed using the WHO’s Global Age-Friendly Cities Guide.	The dimension of outdoor spaces and buildings received the highest score (32.06 ± 11.94), while the dimension of communication and information obtained the lowest score (9 ± 0.27).
Sharqi et al. 2016 [49]	Tehran	Descriptive	Evaluating Status of Global Indices of Age-Friendly City in Tehran Metropolis (AFC)	A total of 110 experts in the field of geriatrics were chosen using convenience sampling and assessed using the WHO’s questionnaire.	The transportation index had the highest score (6.57), while the civic participation and employment index had the lowest score (2.05).
Janipour et al. 2019 [47]	Karaj	Descriptive	Environmental Needs Assessment of the Elderly in Karaj Urban Parks	A total of 299 older adults and their cohabitants were selected using stratified random sampling and assessed employing a standard questionnaire.	The older adults expressed dissatisfaction with the absence of fences and sloping surfaces, memorable elements, cool halls, and relief facilities.
Iranshahi et al. 2017 [14]	Isfahan	Descriptive (case study)	An evaluation of Urban Spaces Conformity with the Indicators of Age-Friendly City; the Case of Chahar Bagh-e Abbasi Street of Isfahan	The geographic information system (GIS) was used to observe, measure, and analyze the desirability of indicators.	Sanitary services, public transportation, and rest areas were identified as the most desirable indicators.
Bastani et al. 2016* [46]	Tehran	Descriptive	Age–friendly Cities Feature from the Elderly’s Perspectives Underscoring “Community Support and Health Services”	A stratified sampling method was used to select 400 older adults and the WHO’s guide for community support and health services was used to assess them.	The total mean score for the community support and healthcare services index was 26.82, falling below the questionnaire’s midpoint. The lowest mean score was linked to free home care services.
Taraghi et al. 2018 [32]	Sari	Descriptive	A Comparison of Older Adults’ and Managers’ Attitudes towards Age-Friendly City Indexes.	A total of 379 older people and 57 managers were randomly selected and assessed using the WHO’s questionnaire of indicators of age-friendly cities.	The older adults expressed concerns about the absence of a dedicated information center to address their health and service needs, as well as the inadequate policy for constructing new housing tailored to their requirements.
Sabouri Garde 2019 [28]	Ardebil	Descriptive (case study)	Design of Urban Spaces for the Elderly and Disabled (Case Study: Shorabil Recreation and Tourism Area)	A total of 381 citizens, were selected through convenience sampling and assessed through a researcher-developed questionnaire, interviews, and field observations.	Upon evaluating the three dimensions of usage, physical aspects, and environmental factors, it was determined that this area lacked ramps, escalators, parking, and benches, making it unfavorable for older adults.
Izanloo et al. 2021 [44]	Tehran	Descriptive	Spatial Justice in the Age-Friendly City Index of Tehran	A multi-stage sampling method was employed to select 770 older adults and the WHO’s standard questionnaire was used to assess them.	Tehran achieved a score of 2.07 out of 4 for the age-friendly city index. Among the indices, the open spaces and buildings index had the highest average (2.33), while the housing index had the lowest average (1.31).
Kiaie et al. 2019 [61]	Qazvin	Descriptive	Evaluating Age-Friendly City Indicators in Qazvin: Urban Open Spaces, Buildings and Public Places	A cluster random sampling method was used to select 200 older adults, while a convenience sampling method was used to select 40 urban managers. They were assessed using the age-friendly city questionnaire based on the WHO’s guide.	The buildings, public places, and open spaces were found to be below the ideal standard for older adults. The mean score of the urban open spaces index was significantly higher than the mean score of the managers.
Araee et al. 2020 [16]	Karaj	Descriptive (case study)	An Analysis of the Experience of the Old Friendly City (Case Study: Karaj City)	Some areas of Karaj city were observed using a researcher-made checklist that assessed physical and social dimensions.	Within the physical dimension, transportation was the least desirable indicator for an age-friendly city. In the social dimension, social security was the least desirable indicator for an age-friendly city.
Khoddam et al. 2020* [33]	Gorgan	Descriptive	The Age–Friendly Cities Characteristics from the Viewpoint of Elderly	A total of 160 older adults were selected using a multi-stage random sampling method and assessed using the WHO’s Age-Friendly City Questionnaire, focusing on 4 indicators.	The highest score belonged to the level of access to social support and health services (81.43), while the lowest score belonged to the transportation index (43.3).
Zaraghani et al. 2015 [15]	Mashhad	Descriptive	Evaluation of Indicators of the Elderly City in Mashhad with Emphasis on Cultural-Social Indicators.	A total of 380 older adults were selected using a stratified sampling method and assessed employing a researcher’s questionnaire based on the WHO’s criteria across four components.	Except for the component of healthcare services, which was reported below the standard, the rest of the indicators were average and slightly higher. The lowest score was related to the health service index.
Soleimani et al. 2012 [41]	Tehran	Descriptive	Ranking of Tehran Metropolitan Areas based on Elderly Life Indicators.	A total of 22 districts of Tehran were selected using the census method and assessed using the Shannon entropy model and the TOPSIS multi-criteria decision-making technique.	In comparison to other districts of Tehran, the East and South districts were found to be in less favorable conditions. District 17 was ranked as the least suitable.
Rakhshaninasab et al. 2022 [35]	Yasuj	Descriptive	Evaluating Indicators of the Desirable City for the Elderly (Case Study: Yasuj City)	A random sample of 380 older adults was surveyed using a researcher-developed questionnaire to assess the elderly-friendly city across five components.	The city lacked appropriate conditions for the elderly, with the access dimension being the most favorable and the transportation dimension being the least favorable.
Nekooei et al. 2021 [45]	Isfahan (Hezar Jarib Neighborhood)	Descriptive	Explanation of the Planning of Elderly-Friendly Urban Spaces, A Case Study: Hezar Jarib Neighborhood.	A random sample of 346 citizens was surveyed using a researcher-developed questionnaire to assess the elderly-friendly city across eight components.	The condition of the indicators was deemed unfavorable from the citizens’ perspective, with only three indicators—open space and buildings, housing, and transportation—being considered favorable.
Saberifar 2023 [38]	Metropolitan cities of Iran (Tehran, Mashhad, Isfahan, Shiraz, and Tabriz)	Descriptive	Evaluating the Results of Policies in the Field of Elderly-friendly Cities in Iranian Metropolises.	A total of 500 citizens were selected using a quota sampling method and surveyed using a researcher-developed questionnaire to assess the elderly-friendly city across eight components.	The indexes’ coefficients were below the median (0.492). In most cities, the space and building index was rated as the least favorable.
Mohammadi et al. 2018 [36]	Sabzehvar	Descriptive	Assessment of the Suitability of the Structure of Mosques in the Light of the Elderly’s Needs.	A total of 30 mosques were selected using convenience sampling and assessed using a researcher-made questionnaire.	The outer and inner doors in mosques were not in the same level, and there were a large number of stairs, no ramps, and inadequate sanitary services and ablutions.
Safa et al. 2024* [42]	Kashan	Descriptive	Examining the status of age-friendly indicators in Kashan	A total of 80 locations in four districts of Kashan city were selected by random sampling method and investigated using the researcher’s checklist.	Open buildings and offices, roads and urban transportation, parks, and public spaces, in terms of being friendly to older adults, were in average condition, and only religious places were in good condition.
Abasian et al. 2023 [37]	Khorasan Razavi Province	Descriptive	Investigating the compliance of teaching hospitals of Khorasan Razavi Province of Iran with the criteria of age-friendly hospitals.	A total of 16 teaching hospitals were selected using census sampling and assessed using the checklist of age-friendly hospitals.	The lack of trained personnel in the field of geriatrics, inappropriate healthcare management systems, and lack of prioritizing older adults in providing services were the main problems of hospitals.
Komasi et al. 2023* [29]	Songhor	Descriptiv-analytical	Evaluating the health-treatment situation of Songhor City about usefulness for the elderly	A total of 30 experts in urban planning, housing, and medicine were selected by snowball sampling and their opinions were analyzed using a researcher-made questionnaire.	About two-thirds of treatment facilities are inaccessible to older adults. Government policies were the most important driving factor for creating an age-friendly city.
Mohammadaghaee et al. 2015 [23]	Karaj	Mixed method	Restoring the Appropriate Model in the Design of Urban Sidewalks with the Approach of Responding to the Needs of the Elderly (Case Study: Jahanshahr Area of Karaj)	A total of 100 older adults were chosen through convenience sampling to participate in interviews and complete a researcher-developed questionnaire focusing on the four components affecting sidewalks.	Lack of suitable space for walking, poor ease of access, interference of vehicular and pedestrian axes, the poor quality of passages, and concerns about social security, had caused older adults to be dissatisfied with the sidewalks.
Kalantar et al. 2021 [22]	Babol	Qualitative	Identifying Structural Suitability Components of Aged-friendly Mosques in Iran: A Qualitative Study.	A total of 18 older adults were selected through purposeful sampling and semi-structured interviews.	The older adults highlighted issues such as inadequate parking access, insufficient public transportation, inadequate safety measures, problems with the cooling and heating systems, and inadequate ablutions and sanitary services.
Sadeghi et al. 2012 [21]	Tabriz	Qualitative	Elderly People and Their Family Care Explanation of Their Experience from Age–Friendly City of Tabriz	A total of 32 older adults or their caregivers were selected through purposeful sampling and group interviews.	The most unpleasant experiences included the absence of a sewage system, the presence of vermin, inadequate transportation, and issues related to addicts in the parks.

* The text of articles are in English

The systematic review revealed that, apart from two qualitative studies [21, 22] and one quantitative-qualitative study [23], the remaining studies were descriptive. Geographically, seven studies were conducted in the west of Iran [21, 24–29], five in the north [22, 30–33], two in the south [34, 35], three in the east [15, 36, 37], one in several geographical regions [38], and others in the central region of Iran. The most commonly used tool across the studies was the WHO’s Global Age-Friendly Cities Guide. Data in some studies were collected through observation [14, 16, 36, 37, 39–42], while others gathered data from the perspectives of older adults, citizens, managers, or experts in the field of geriatrics.

According to the WHO’s indicators of the age-friendly city, only eight studies investigated all eight indicators [25–27, 30, 31, 38, 43–45]. Some studies focused on specific indicators, while others investigated areas not included in the eight standard indicators. Additionally, some studies examined entire cities, while others focused on specific districts within a city on a case-by-case basis [14, 28, 45]. Certain studies specifically addressed hospitals and healthcare services [29, 37, 39, 40, 46], mosques [22, 36], sidewalks [23], and parks [47].

Out of the 34 reviewed studies, only two studies in Tehran and Mashhad provided some insight into the condition of the indicators about the needs of older adults [15, 44], while other studies reported unfavorable urban conditions for older adults. Despite these unfavorable conditions in many Iranian cities, the following ranking was obtained among the indicators examined in the studies:

The index of open spaces and public buildings exhibited a better status than other indices in five studies [27, 31, 35, 44, 45]. In a study in Zanjan, the physical contribution index had a better condition than other indices [48]. In a case study, access to restrooms and sanitary services was identified as the most desirable indicator [14].

The transportation index [30, 38, 49] and the social support and health services index [12, 24, 33] were better than other indices in three studies. The housing index [13, 34], social participation index [26, 34], and communication and information index [15, 25] were in better condition than other indices in the two studies. The recreational cultural index was better than other indices in only one study [15]. However, the two indicators of respect for older adults and social acceptance, and civic participation and employment, did not have a good status in any study compared to other indicators.

In three studies that exclusively focused on hospitals, it was found that the most favorable indicators of hospitals and healthcare systems included the safety of the physical environment [39, 40], access to transportation [39], and respectful behavior of the staff, particularly towards older adults [46]. However, resource management, specialized programs for older adults, their training and prioritization, and free home care services were not in good condition [39, 40, 46].

Two studies exclusively examined mosques and identified issues such as inadequate access to parking, lack of public transportation, gender inequality in the division of interior space of mosque, inadequate safety measures, problems with the cooling and heating systems, lighting and ventilation, and insufficient ablutions and improper sanitary services were among the concerns reported by older adults [22, 36]. The major defects in the eight dimensions of the age-friendly city are mentioned in Table 3.

**Table 3:** The major defects in the eight dimensions of the age-friendly city in Iran

Domains of age-friendly cities	Major defects
The open spaces and public buildings	-Inappropriate traffic design (Fig. 3) [30] -Narrow sidewalks with unsafe flooring (Fig. 4) [14, 30] -Lack of standard furniture and equipment for older adults (Fig. 5) [21, 49] -Lack of proper fences and ramps [14, 21, 23, 28] -Lack of escalators [49] -Lack of spaces with heating systems and emergency services in open spaces [47]
The transportation	-The unavailability of transportation services at all hours of the day [25] -The improper public transportation equipment [16, 30, 42] -The lack of special parking spaces for older adults [28, 37, 42] -The lack of free transportation services for older adults [21] -The lack of special chairs for older adults’ public transportation [13, 24]
The social support and health services	-The lack of homecare services [33, 34] -Lack of support services at health centers for older adults [39] -Financial barriers to using health services [13, 46] -Lack of specialized programs for older adults [37] -Lack of prioritization of services for older adults [12, 21, 37]
The housing	-The lack of policies for age-friendly housing [32] -Inadequate housing safety [13, 21, 25] -Lack of affordable homes for low-income seniors [13, 34]
The social participation	- Older adults lack of participation in urban decision-making [48] - Limited involvement of older adults with community activities [30] - Limited participation of older people in voluntary activities [34]
The communication and information	-Lack of educational programs for older adults [24, 26] -The lack of suitable electronic equipment, including ATMs [24, 30] -The lack of access to information through computers and the Internet in public places [15, 26, 32] -lack of education on new technology for older adults [15, 26] - Lack of information boards with fonts and colors suitable for older adults [26]
The recreational cultural	-Lack of suitable programs for leisure [49] -Lack of suitable sports places [42] -Improper platform for the participation of older adults in cultural affairs [32] -Lack of morning sports programs in parks [24] -Lack of pilgrimage and recreational trips [24]
Respect for the older adults and social acceptance	-Lack of voluntary services for older adults [32] -Lack of participation in social programs [34] -Lack of special shopping mall for older adults [26]
Civic participation and employment	-Lack of suitable job opportunities for older adults [12, 30, 34] -Unsafe workplaces for older adults [49] -Lack of part-time jobs for older adults [44]

## Discussion

The findings of this study indicated that Iranian cities failed to meet the standards of an age-friendly city. Out of the reviewed studies, only two, conducted in Tehran and Mashhad, partially addressed the indicators in line with the needs of older adults, while other studies reported unfavorable urban conditions for older adults. Aging is an important stage of life that cannot be stopped. Therefore, it is crucial for urban policy and development to realize the indicators for an age-friendly city as much as possible. Given the increasing number of older Iranians, it is recommended that policymakers in the field of geriatric health take steps to identify and mitigate environmental risk factors for older adults.

The results of the systematic review revealed that among the eight indicators of an age-friendly city, open spaces and public buildings exhibited a better status than other indicators. Open spaces and public buildings serve as places for both group and individual activities, where people spend part of their leisure time. These spaces are vital urban infrastructures. Tailoring these spaces to the specific needs of older adults can facilitate and enhance social interactions and communication among them, leading to improved physical and mental well-being and an enhanced quality of life. The availability of public spaces for all individuals promotes social equity [50]. A study in China demonstrated that the presence of parks and well-lit environments had the greatest impact on the activities of Chinese older adults. This study also found that there was insufficient support for the specific activities of older adults within welfare buildings [51]. Another study indicated that uniform, glare-free lighting in open environments could increase the utilization of public spaces by creating a sense of security and maintaining safety [52].

In the current research, older adults expressed concerns about issues such as improper traffic, conflicts between pedestrian and vehicular pathways, poor quality sidewalks, the presence of pests in public spaces, inadequate bus routes, lack of proper ramps and handrails, absence of escalators, inadequate parking, seatings, and relief facilities in parks. Given that municipalities are the primary custodians of these spaces in Iran, it is essential to communicate the shortcomings identified in the studies to them to address these issues.

The results of this systematic review revealed that, among the eight indicators of an age-friendly city, two indicators—respect for older adults and social acceptance, and civic participation and employment—did not exhibit a favorable status in any study compared to other indicators. In most of the studies, not all eight indicators of the age-friendly city were investigated possibly due to the challenges associated with measuring these two indicators. Thus, researchers have devoted less attention to them and focused more on examining more objective indicators such as the physical environment.

Respecting human dignity is one of the most crucial needs of older adults, emphasized in the rich Iranian culture and Islam, which promotes respect for older adults in society and recognizes them as a source of goodness and blessing to the community [43]. There are conflicting results in the studies regarding the employment of older adults. For instance, a study in China found that working after retirement had a negative effect on mental health in old age [53]. Another study considered old age, poor health, and negative attitudes toward older adults as reasons for not employing them in various jobs [54]. However, society should provide a suitable platform for older adults who, for various reasons, wish to continue working in their old age.

In the current research, certain studies focused solely on the healthcare systems for older adults, particularly hospitals. Strengths included the safety of the environment, access to transportation, and respectful behavior of hospital personnel towards older adults. Weaknesses encompassed mismanagement of resources, absence of specialized programs for older adults, inadequate training and prioritization of their needs, and a lack of free home care services within these systems. A study in Canada revealed that emergency departments were not physically suitable for older adults, citing issues such as overcrowding, lack of directional signs, and inadequate equipment and seating [55].

Healthcare systems are not solely responsible for diagnosing and treating diseases; they must provide social and environmental conditions that cater to the needs of older adults, including social participation, transportation, safety, and the dissemination of educational information to promote healthier behaviors. In Iran, weaknesses in healthcare systems are linked to inadequate regulatory structures and standards, insufficient government commitment, weak regulations and structures, and inconsistencies between wards and professions. Home care, a critical need for older adults within Iran’s healthcare system, necessitates the implementation of policies related to culture, time investment, and adequate resources, as well as the training of specialized human resources for home care [56]. Given the increased prevalence of chronic diseases among older adults, it is imperative to adapt healthcare systems to meet their specific needs.

Mosques are crucial urban environments and buildings frequently utilized by older adults. Some studies included in the current review also examined mosques. The inadequacy of the physical environment in mosques has undermined the accessibility and safety of these spaces for older adults. In the cultural and religious context of Iran, older adults have a strong inclination to attend religious centers, including mosques. In addition to fulfilling spiritual needs, these places can serve as centers for communication, social participation, and leisure time activities [57]. This aspect fosters self-esteem and a sense of value in older adults. Therefore, these spaces should be tailored to accommodate the needs of older adults.

Culture and religion have a significant impact on shaping the experiences of older adults in Iran. Iranian culture emphasizes respect for elders. Older people are often seen as a source of wisdom and experience, and their opinions and contributions are highly valued [58, 59]. In light of this culture, people and authorities should try to design and implement age-friendly cities in Iran. For example, public spaces and infrastructure often need to be created with respect for the specific needs of older adults in mind, such as providing accessible transportation, well-maintained sidewalks, and public seating areas. Additionally, religion, predominantly Islam, plays an important role in shaping the experiences of Iranian older adults. Islamic teachings emphasize the importance of caring for the elderly and promoting social cohesion within communities. This religious influence promotes a sense of community support and encourages the provision of health services, social activities, and spiritual support tailored to the needs of older adults [60]. Generally, culture and religion in Iran can contribute to creating age-friendly cities by prioritizing the well-being, inclusion, and respect of older adults through various indicators and initiatives.

In the reviewed studies, the index of open spaces and public buildings ranked higher than other indices. Following that, the transportation index, and social support and health services index held the second position, while the housing index, social participation index, and communication and information index occupied the third position. The cultural and recreational index ranked fourth. The two indicators of respect for older adults and social acceptance, and civic participation and employment did not perform well in any study compared to other indicators. Addressing the needs of the Iranian older adults and enhancing their participation in citizenship affairs will enhance the presence of older adults in society and promote their social spirit.

According to the defects in the areas of respect for older adults and social acceptance, and civic participation and employment, the following suggestions to the authorities and policymakers are presented: creating special queues for older adults in public offices and centers, giving priority to the employment of older adults in centers such as museums that are suitable for their physical conditions and on the other hand use their valuable experiences, using the capacity of older adults in urban management and assigning the management of charitable institutions to older adults, establishing daily clubs for older adults, holding commemoration and appreciation meetings for older adults on various cultural and religious occasions.

Also, the following things are recommended to the authorities and policymakers to make open spaces and public buildings more compatible with the conditions of older adults: improving transportation conditions for older adults, establishing special parking spaces for older adults, increasing environment safety to support the participation of older adults in social activities, requirements for obtaining an elderly safety certificate for newly constructed and remodeled buildings.

Iranian cities are facing challenges to becoming age-friendly cities. For example, many cities are historical, and making changes in them will face obstacles that may interfere with the recommendations of the country’s cultural heritage to preserve these historical monuments. Due to the growing elderly population in Iran, issues of older adults have become of increasing interest to managers and policymakers in the field of health and aging. This can be seen as an opportunity to promote Iran’s urban policy to solve the problems of older adults.

This study represented the first systematic review of Iranian cities based on age-friendly city indicators. It is anticipated that policymakers in the field of aging in Iran can utilize the findings of this study to enhance the well-being of Iranian older adults.

### Limitations

It is important to note some limitations of this study such as the exclusion of articles published in languages other than Persian, the omission of interventional studies, and the inability to access the full texts of certain articles despite attempts to contact the authors. These limitations should be taken into account in future research attempts.

## Conclusion

Aging is an inevitable part of life for every individual. The findings of this study indicated that Iran failed to meet the required standards for older adults. Among the reviewed studies, the index of open spaces and public buildings ranked higher than other indices. However, the indicators of respect for older adults and social acceptance, as well as civic participation and employment, did not perform well in any study compared to other indicators. Therefore, it is imperative to give special attention to these two indicators.

Creating age-friendly cities in Iran can contribute to the well-being and safety of Iranian older adults and allow them to actively participate in society regardless of their abilities. In light of the increasing older population in Iran, it is recommended that policymakers in the field of elderly health take proactive measures to identify and mitigate environmental risk factors, thereby enhancing the quality of life for older adults and promoting urban social justice. The results of this research serve as a call for policymakers to take appropriate action. Policymakers should pay special attention to this issue and assign more budgets to make the cities safe by creating a suitable platform for increasing the social integration of Iranian older adults. The creation of age-friendly cities necessitates an interdisciplinary approach. Therefore, engineers, architects, geographers, urban planners, geriatric experts, nurses, healthcare managers, and sociologists should collaborate, align their efforts, and focus on both short-term and long-term goals to achieve the desired standards in this area.

### Registration and Protocol

This systematic review was registered on the Prospero database with the number CRD42023475657 on date 8 November 2023.

## Data Availability

The data of this research are available on request from the corresponding author. The protocol of this systematic review is available on the Prospero database with the number CRD42023475657 on date 8 November 2023.

## Abbreviations

WHO: World Health Organization
PRISMA: Preferred Reporting Items for Systematic Reviews and Meta-Analyses
MeSH: Medical Subject Headings
GIS: Geographic Information System
TOPSIS: Technique for Order of Preference by Similarity to Ideal Solution
ATM: Automated teller machine
